# Effects of Kindlin-2 on proliferation and migration of VSMC and integrinβ1 andβ3 activity via FAK-PI3K signaling pathway

**DOI:** 10.1371/journal.pone.0225173

**Published:** 2020-06-30

**Authors:** Xiaolin Wu, Fang Bian, He Hu, Tongjian Zhu, Chenyu Li, Qing Zhou

**Affiliations:** Department of Cardiology, Xiangyang Central Hospital, Affiliated Hospital of Hubei University of Arts and Science, Xiangyang, Hubei, P.R. China; Duke University School of Medicine, UNITED STATES

## Abstract

Vascular hyperplasia after vascular trauma is one of the difficult problems in clinical treatment. Nowadays, there is no effective treatment for vascular hyperplasia. Previous studies have shown that integrinβ1 andβ3 activity play an important role in vascular hyperplasia. Kindlin-2 has been shown to modulate integrinβ1 andβ3 activity in cancer. Therefore, in this study, we hope to explore the relationship between Kindlin-2 and vascular hyperplasia. We overexpressed or knocked down Kindlin-2 by adenovirus. The results showed that Kindlin-2 overexpression could regulate integrinβ1 andβ3 activity through FAK-PIK3 signaling pathways ex vivo and in vivo, thereby affecting the proliferation and migration of VSMC, and then it causes the consequences of vascular hyperplasia. Therefore, Our results show that Kindlin-2 may be a potential target for the treatment of vascular hyperplasia.

## Introduction

Intimal hyperplasia is a typical feature after vascular injury. Endometrial hyperplasia is a chronic vascular wall structural change, accompanied by a variety of inflammatory factors, cytokines, chemokines secretion, thereby promoting vascular smooth muscle cell(VSMC) migration, proliferation, and secretion of extracellular matrix of a pathophysiological process [1]. After arterial injury, the blood vessels will respond, which will last for weeks or even months. During this process, intimal hyperplasia will occur. This process mainly involves 3 factors: vascular smooth muscle cells, endothelial cells and extracellular matrix [2]. Among them, the proliferation and migration of vascular smooth muscle cells are the main pathological features of intimal hyperplasia.

Vascular smooth muscle cell proliferation is evident in 24h after arterial injury and last for at least 2 weeks [3]. At the same time, VSMC, stimulated by TGF-b, PDGF and other factors, secreted a large number of extracellular matrix and accumulated, forming vascular lumen stenosis and occlusion.

In clinic, in addition to external injury, cutaneous artery angioplasty, stent implantation, vascular bypass grafting and autologous vein grafting can cause intimal hyperplasia, leading to surgical failure. At present, the specific mechanism of vascular hyperplasia is still under exploration. However, the proliferation and migration of vascular smooth muscle cells is one of the important causes of vascular hyperplasia [4].

Many factors, such as Wnt, AKT and FAK, contribute to the migration and proliferation of vascular smooth muscle cells. In previous studies, researchers have found that vascular proliferation is also associated with integrin activity. Previous studies have shown that classical FAK-PI3K-AKt signaling pathway can activate Integrin activity [5]. Integrin can mediate the connection between extracellular matrix and intracellular signaling pathways. In the process of vascular hyperplasia, integrin will further enhance this process when vascular smooth muscle migrates and proliferates [6,7].

Kindlin-2, also known as FERMT2, is a scaffolding protein that enhances integrin activation. In particular, Kindlin-2 can enhance integrin mediated cell adhesion and migration [8]. In previous studies, kindlin-2 promoted the invasion and migration of different tumor cells through AKT and NF-kb pathways in various cell lines [9–11]. In particular, kindlin-2 also could promote the migration of gastric cancer cells by promoting phosphorylation of integrin beta 1 and beta 3 in gastric cancer cell lines [12]. However, although the effect of kindlin-2 is coincident with the mechanism of promoting vascular hyperplasia, the role of kindlin-2 in vascular hyperplasia has not been studied. Therefore, our study aims to explore the role of kindlin-2 in vascular hyperplasia by using rat vascular smooth muscle cells and rat vascular injury and hyperplasia animal model.

## Materials and methods

### Antibody

In this experiment, we used a variety of antibodies in Western and immunofluorescence, including: anti-kindlin-2(SIGMA), anti-FAK, anti-AKT (Proteintech Group, Wuhan, China), ati-P-AKT, anti-P-FAK(CST), anti-ITGFβ1, anti-ITGFβ3(Millipore), anti-P- ITGFβ1(Bioworld), anti-P- ITGFβ3(NOVUS), anti-Brdu(CST), HRP labeled Goat anti mouse antibody, HRP labeled Goat anti rabbit antibody and HRP labelled rabbit against rat antibody(Boster Biological Technologyco, Wuhan, China).

### Isolation of rat aortic smooth muscle cells

The rats were killed, then opened chest and chest skin. The aorta vessels of rats were removed and placed in a pre cooled DMEM medium. The vascular medium membrane was separated under a microscope. The separated middle membrane was cut and broken by surgical scissors and cultured in DMEN with 20% fetal bovine serum.

### Identification of rat aortic smooth muscle cells

When the degree of fusion of vascular smooth muscle cells reached about 90%, anti-α-SMA antibody was used to identify whether the isolated primary cells were vascular smooth muscle cells by immunofluorescence staining.

### Cell culture

The VSMC cells were cultured using DMEM medium supplemented with 20% of fetal bovine serum, 1% sodium pyruvate (100 mM), 1% non-essential amino acids (10 mM) and 1% penicillin/streptomycin solution (Invitrogen, Carlsbad, CA). Cells were maintained at 37°C at 5% CO2 atmosphere. The cells were passaged every 5 days.

### Immunofluorescence

For immunofluorescence, cells were plated on chamber slides, fixed either with methanol at −20°C for 5 min or with 4% paraformaldehyde at 37°C for 15 min depending on the antibodies used. To examine the protein levels at each mitotic stage, cells were synchronized by double-thymidine block and release to fresh media for various times. A staging system was used to identify the different phases of mitosis and cytokinesis based on the DNA and spindle morphology and extent of chromosome alignment and separation. To test the stability of Microtubule capture at kinetochores, cells were incubated for 5 min on ice before fixation, to destabilize most non-kinetochore Microtubules. After fixation, cells were permeabilized with 0.2% Triton for 5 min, preincubated with centrifuged (14,000 rpm) supernatant of 5% FBS and 5% goat serum in PBS and incubated with primary antibodies overnight. Slides were washed, incubated with fluorescence-tagged secondary antibodies (Alexa Fluor 488, 568 and 647, Molecular probes, Invitrogen), and counterstained with DAPI (Vector Labs) for 1 h at 4°C. Cells were visualized and imaged using a Zeiss LSM710 confocal microscope equipped with a ×60 objective. All immunofluorescence experiments were conducted at least three times. Image processing and figures were made using PhotoShop CS (Adobe). Images of proteins of interest as well as CREST on kinetochores were acquired by using identical imaging settings.

#### Immunohistochemistry

Selected sections were deparaffinized, rehydrated, and heated in a microwave oven in 0.01 M citrate buffer (pH 6.0; Química Contemporânea, Diadema, Brazil) for 30 min. Endogenous peroxidase activity was blocked by 3% hydrogen peroxide for 10 min, followed by a wash with phosphate buffered saline. The sections were incubated overnight at 4°C with the antibodies. The antibody was then detected using avidin-biotin peroxidase detection solution (Dakocytomation labelled streptavidin biotin reagent; Dakocytomation, Glostrop, Denmark and System-horseradishperoxidase; Dako, Glostrup, Denmark) and the signal was visualized using diaminobenzidine (Dakocytomation) and Substrate Chromogen-System (Dako). Slides were counterstained with Harris’s hematoxylin, dehydrated, cleared and mounted. Positive controls from the appendix and tonsils were used. The cells were initially observed at a low magnification (×100) to assess the general distribution of the antibody. The samples were subsequently examined at a higher magnification (×400). The evaluation of cell staining was performed in tumor tissue. The tumor cells (exhibiting gross and evident nucleoli, and irregular chromatin) were identified and counted at the higher magnification.

### Western blot

Cells were harvested, lased, and subject to SDS-PAGE gels, which were prepared depending on the protein size. Electrophoresis and transmembrane were carried out on a protein electrophoresis and blotting system (Liuyi, Beijing, China), followed by the incubation with primary antibody. Membranes were visualized using the Immun-Star WesternC Chemiluminescence Kit (Liuyi, Beijing, China) and images were captured using a ChemiDoc XRS+ System and processed using Image Lab software (Liuyi, Beijing, China).

### Real-time quantitative PCR

cDNA templates were prepared after RNA extraction and reverse transcription. Amplification was performed on a real-time PCR system (Applied Biosystems 7500, USA). The whole procedure was according to the manual of SYBR® Premix Ex Taq™ kit (Takara RR420A, Japan). Relative expression was calculated using the formula of 1/2△Ct. The primers for detecting differentiation related gene were as follows: β-actin, forward 5‘-CACGATGGAGGGGCCGGACTCATC-3’, reverse 5‘- TAAAGACCTCTATGCCAACACAGT-3’; C-myc, forward 5‘- CGAGCTGAAGCGTAGCTTTT-3, reverse 5‘- CTCGCCGTTTCCTCAGTAAG-3; CyclinD1, forward 5‘- TGCCACAGATGTGAAGTTCATT-3, reverse 5‘- AGAAGGGCTTCAATCTGTTCC-3.

#### Transwell assay

After the logarithmic growth of VSMC cells were digested and washed with PBS, the cell was suspended with 3%FBS DMEM, and the cell density was adjusted to 5 x 10^5^ /ml. The cell suspension containing different concentrations of 200 ul was added to the Transwell upper compartment. The 500 μl DMEM medium with 10% FBS was added to the lower compartment and cultured for 12 hours in 37°C and 5% CO2 incubator. Cells were fixed with Formaldehyde and dyed with Giemsa then observed and photographed under a microscope.

#### Ethical statement

This study was approved by the institutional review board (CWO) of Affiliated Hospital of Hubei University of Arts and Science.

### Statistical analysis

Statistical analysis was performed using SPSS 17.0 software. The differences were evaluated by using a two-way Anova or t-test. Error bars represent standard errors (SD), and three independent experiments were performed for each assay. Statistical significance was indicated at *P<0.05, **P<0.01 or ***P<0.001. All statistical analyses and comparisons were made between the experimental groups and the control group. Histograms were drawn using GraphPad Prism 5.01, and every figure was combined using Photoshop CS6.

## Results

### Isolation and identification of rat vascular smooth muscle cells

**Fig 1:**
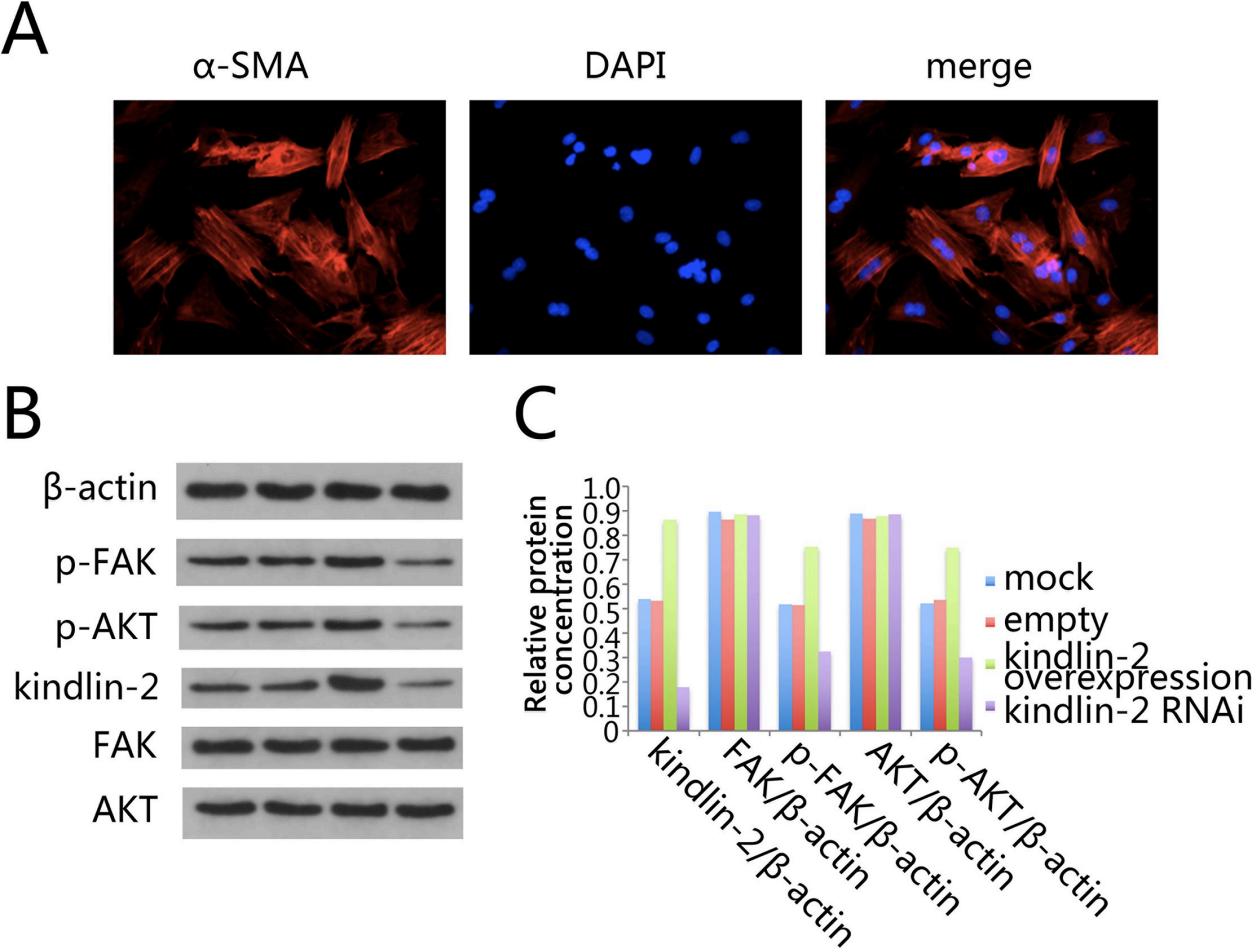
The role of Kindlin-2 in FAK and AKT proteins in VSMC. (A) Identification of VSMC isolated from rats. The cells were isolated from rat vascular smooth muscle and cultured stably and identified by immunofluorescence staining against α-SMA. (B) Total protein was extracted from the cells of different treatment groups, and then Kindlin-2, FAK, AKT, p-FAK and p-AKT were detected by Western blot. From left to right were the control group, the mock group, the Kindlin-2 overexpression group and the Kindlin-2 RNAi group. (C) The expression of Kindlin-2, FAK, AKT, p-FAK and p-AKT in Western blot experiment was quantitatively analyzed by software.

The rats were sacrificed after one week's adaptation to the environment. The vascular smooth muscle was isolated from rats as described previously [13]. VSMC was cultured until the cells grew to about 90%. After obtaining enough cells, we identified the obtained cells in order to identify whether they were VSMCs. Because α-SMA belongs to one of the basic components of cytoskeletal microfilaments and is specifically expressed in VSMC cells, α-SMA can be used as a biomarker of VSMC cells [14]. We identified the α-SMA in the cells by immunofluorescence. The results showed that all the cells we isolated were highly expressed in α-SMA, which indicated that the cells we isolated were VSMC (Fig 1A).

### The role of kindlin-2 knockdown or over expression in FAK-PI3K in VSMC

Activation of FAK-PI3K signaling pathway has been reported to play a key role in the proliferation of damaged blood vessels [5]. And kindlin-2 has proved that it can affect the activity of FAK-PI3K signaling pathway and AKT protein is a typical downstream protein of PI3K signaling pathway [8–10]. Therefore, we want to understand the impact of kindlin-2 on AKT and FAK in VSMC. We first constructed a kindlin-2 knockdown or overexpression adenovirus. After infection with VSMC by adenovirus, we detected the protein expression levels of Kindlin-2, AKT, FAK, p-AKT and p-FAK by Western blot. The results showed that either kindlin-2 knockdown or overexpression had no effect on the expression of AKT and FAK. However, knocking down or overexpression of kindlin-2 can inhibit or promote the phosphorylation of AKT and FAK, respectively (Fig 1B and 1C). This suggests that Kindlin-2 can also affect FAK-PI3K signaling pathway by affecting the phosphorylation of AKT and FAK proteins in VSMC. Therefore, Kindlin-2 may be a potential target for the proliferation and migration of VSMC.

### Kindlin-2 can affect the proliferation and migration of VSMC through FAK-PI3K signaling pathways

**Fig 2:**
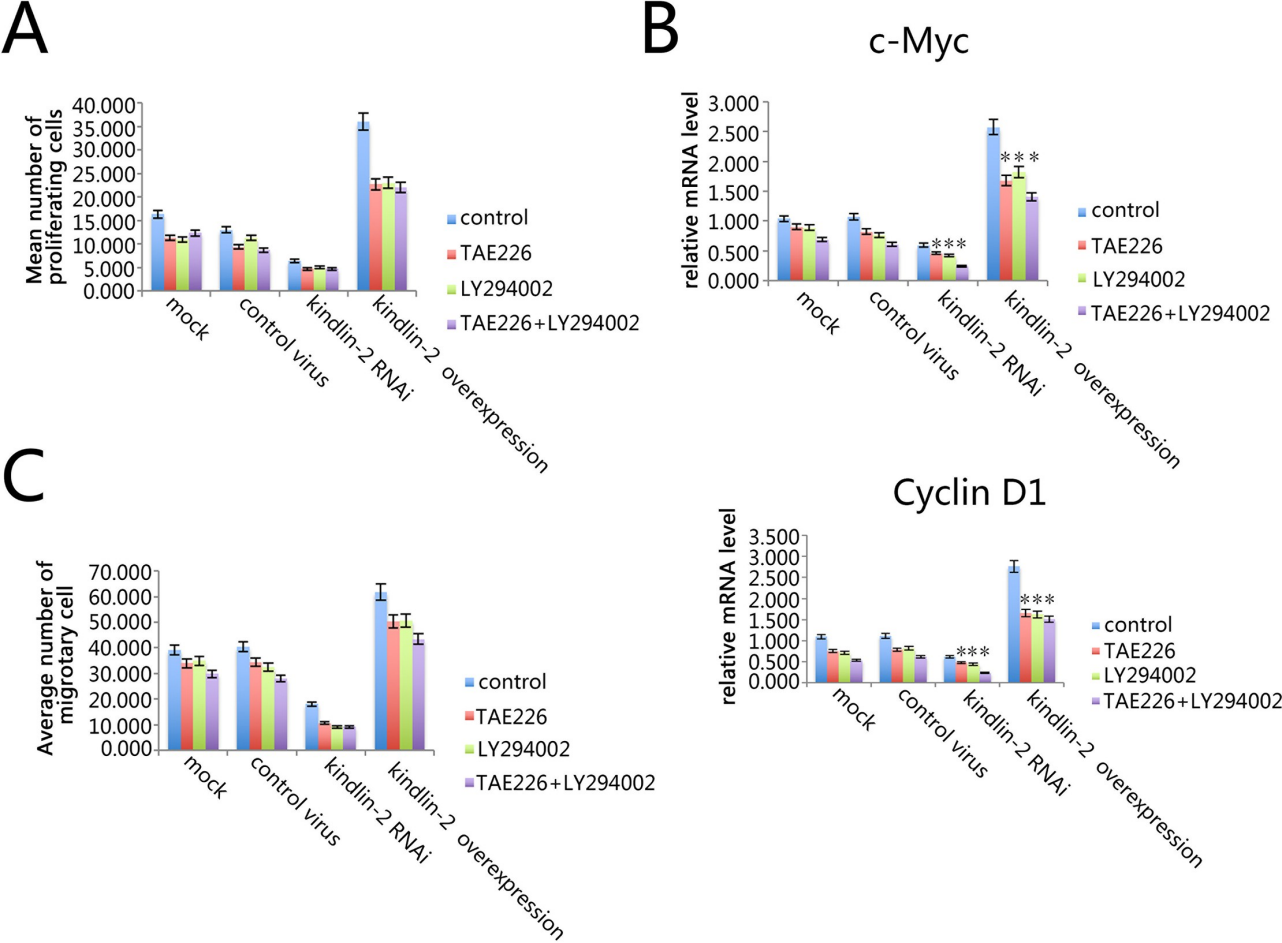
The effect of Kindlin-2 on the proliferation and migration of VSMC cells. (A) Cell proliferation was detected by BrdU assay. The proliferation of VSMC in control group and mock group was almost unchanged. Kindlin-2 RNAi and overexpression could inhibit and promote the proliferation of VSMC, respectively. FAK and AKT inhibitors can inhibit the proliferation of VSMC cells. (B) The expression of proliferation related genes c-Myc and Cyclin D1 was detected by qPCR assay. The mRNA level of c-Myc and Cyclin D1 was detected by qPCR in VSMC of different treatment groups. The experiment was repeated 3 times. *, p < 0.05 (C) Transwell assay was used to detect the migration ability of VSMC. VSMC in different treatment groups was cultured in transwell, and the number of migratory cells was detected under microscope. Kindlin-2 RNAi and overexpression inhibit and promote the migration of VSMC cells, respectively, while FAK and AKT inhibitors inhibit the migration of VSMC cells.

Because in vascular hyperplasia, the most important step are the proliferation and migration of VSMC. Therefore, we decided to further test the role of Kindlin-2 in the proliferation and migration of VSMC. As shown in Fig 1B and 1C, Kindlin-2 affects the level of phosphorylation of AKT and FAK. Therefore, we decided to explore the effect of AKT and FAK inhibition on VSMC and its correlation with Kindlin-2. We first detected the proliferation of cells by BRDU assay. The results showed that over expression of Kindlin-2 could promote the proliferation and apoptosis of VSMC and knocking down Kindlin-2 can promote the apoptosis of VSMC. At the same time, both FAK inhibitor TEA226 and AKT inhibitor LY294002 could promote the apoptosis of VSMC in the Kindlin-2 overexpression, normal expression and knockdown groups. In addition, the effection is more obvious when combined use of TEA226 and LY294002 (Fig 2A). After that, we further detected the proliferation related genes by qPCR. We found that at the level of mRNA, the expression level of Cmyc and Cyclin D1 was negatively correlated with the level of VSMC apoptosis (Fig 2B). Finally, vascular hyperplasia also involves cell migration. So we also detected the migration ability of various treatment groups. The results showed that Kindlin-2 was closely related to cell migration. By inhibiting FAK and AKT, Kindlin-2 can promote cell migration (Fig 2C).

### Kindlin-2 promotes integrinβ1 andβ3 expression in VSMC

**Fig 3:**
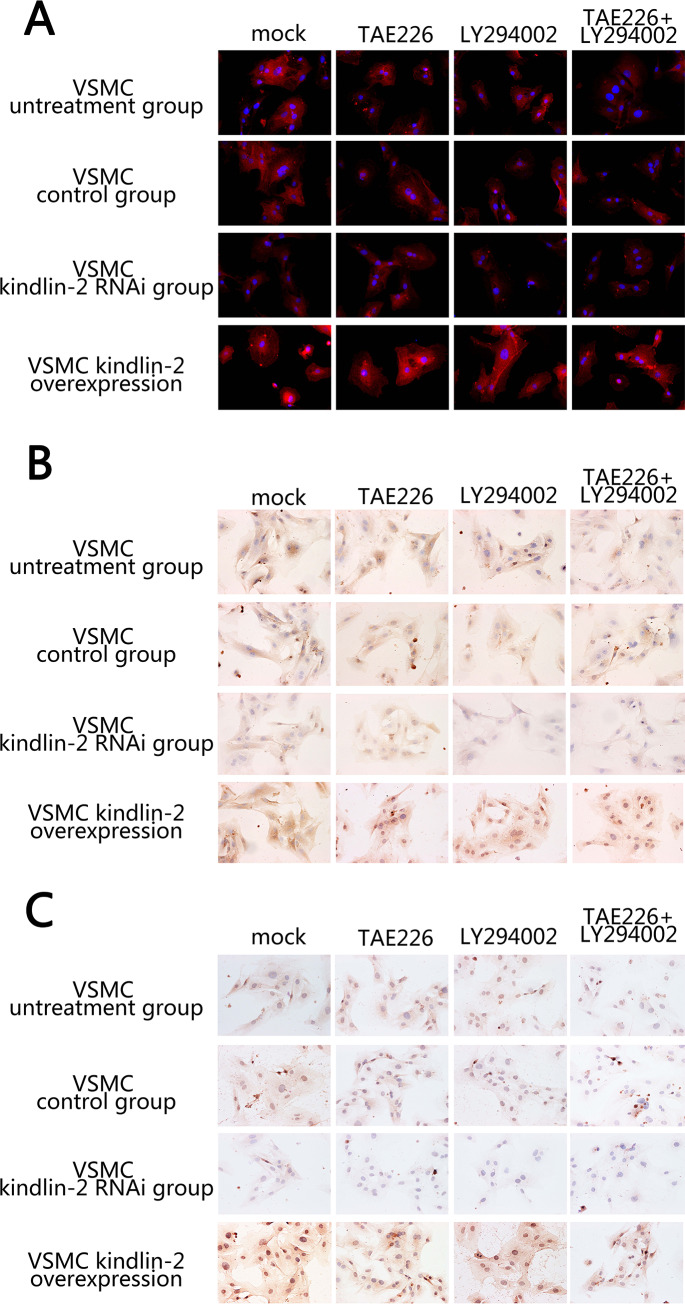
The effect of Kindlin-2 on integrinβ1 andβ3 expression. (A) The expression of Kindlin-2 in VSMC was detected by immunofluorescence. Kindlin-2 RNAi and overexpression of adenovirus could significantly affect the expression of Kindlin-2. However, FAK and AKT inhibitors could not affect the expression of Kindlin-2. (B-C) The effect of Kindlin-2 on integrinβ1 (B) andβ3 (C) expression by immunohistochemistry. Kindlin-2 RNAi and overexpression can inhibit and promote integrin β1 andβ3 expression. At the same time, FAK and AKT inhibitors can inhibit integrinβ1 andβ3 expression in VSMC.

Previous studies have reported that kindlin-2 promotes the recognition and expression of integrins in cancer, and integrins are associated with vascular hyperplasia [12,13]. Therefore, we decided to explore the correlation between kindlin-2 and integrinβ1 andβ3 in VSMC. First, we detected the expression of Kindlin-2 in different groups of cells by immunofluorescence. The results showed that the over expression and knockdown of Kindlin-2 were consistent with expectations (Fig 3A). After that, we detected the expression of integrinsβ1 andβ3 in different treatment groups by immunohistochemistry. The results showed that Kindlin-2 could significantly promote the expression of integrinβ1 andβ3 in VSMC, and could destroy the promotion of Kindlin-2 by inhibiting the FAK and AKT pathways (Fig 3B).

**Fig 4:**
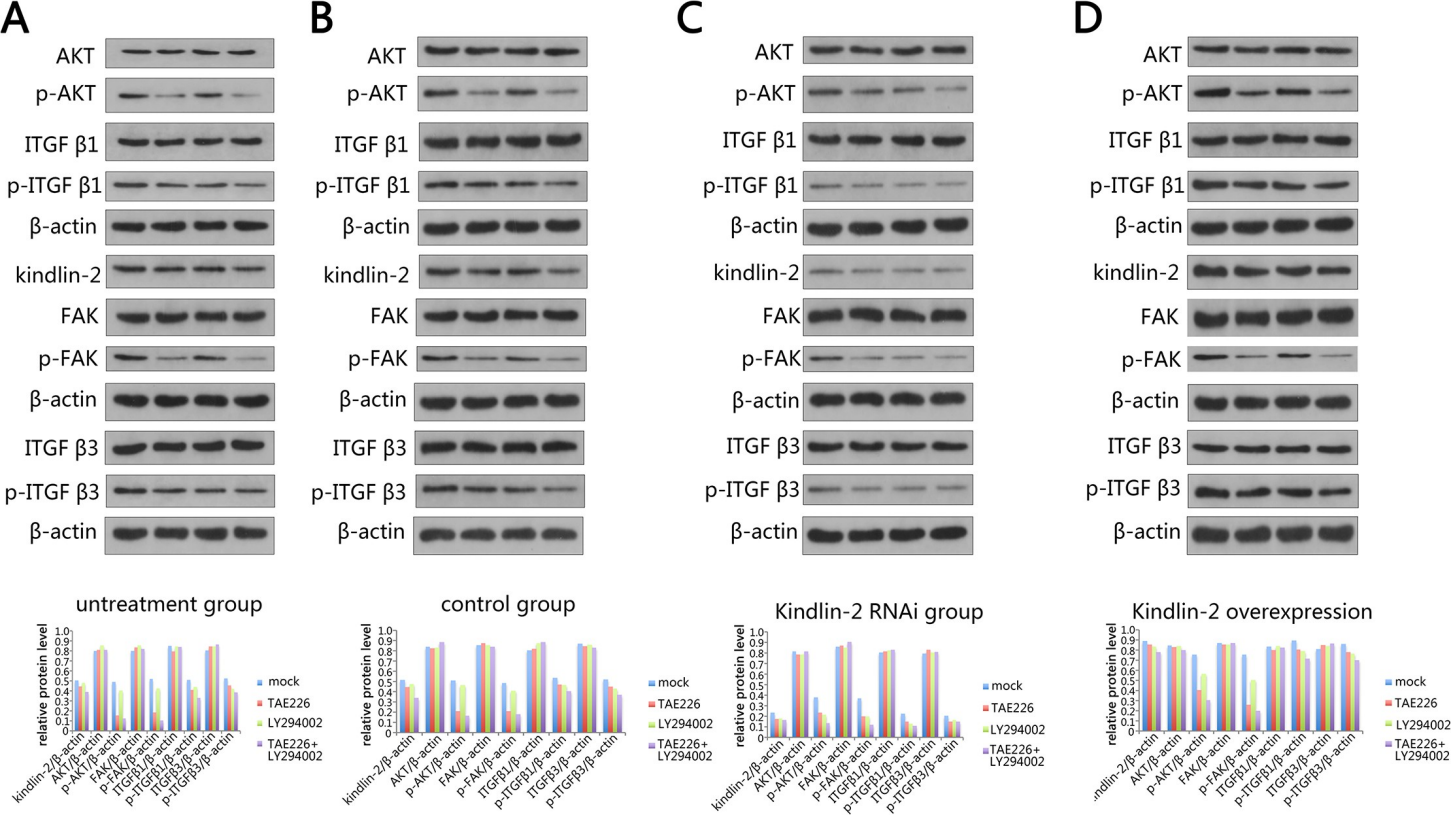
The protein expression levels of Kindlin-2, FAK, AKT and integrinβ1 andβ3 were detected by Western blot. (A-D) Detection of protein expression in untreatment (A), control (B), Kindlin-2 RNAi (C) and Kindlin-2 overexpression (D) groups. We detected the expression levels of Kindlin-2, FAK, p-FAK, AKT, p-AKT integrinβ1, p-integrinβ1, integrinβ3 and p-integrinβ3 in different treatment groups. Gray scale quantitative analysis by software showed that phosphorylation levels of related proteins in different treatment groups increased significantly.

After that, we further analyzed the relationship between Kindlin-2, FAK, AKT and integrinβ1 andβ3 in VSMC by Western. Normally, inhibition of AKT and FAK pathways did not affect the overall expression levels of AKT, FAK and integrinβ1 andβ3 proteins. However, phosphorylated AKT, FAK and integrinβ1 andβ3 decreased significantly, suggesting that Kindlin-2 may play a role by influencing the phosphorylation of these proteins (Fig 4A). We also observed the same phenomenon in the empty virus infection group (Fig 4B). But in Kindlin-2 knockdown VSMCs, we found that Kindlin-2 knockdown did not affect the expression of AKT, FAK and integrinβ1 andβ3 proteins. Kindlin-2 itself lowered the phosphorylation levels of AKT, FAK and integrinβ1 andβ3 proteins. After treatment with AKT and FAK inhibitors, the phosphorylation levels decreased more significantly (Fig 4C). In the Kindlin-2 overexpression group, we just got the opposite result (Fig 4D).

### Confirming the role of Kindlin-2 in rat models of vascular hyperplasia

**Fig 5:**
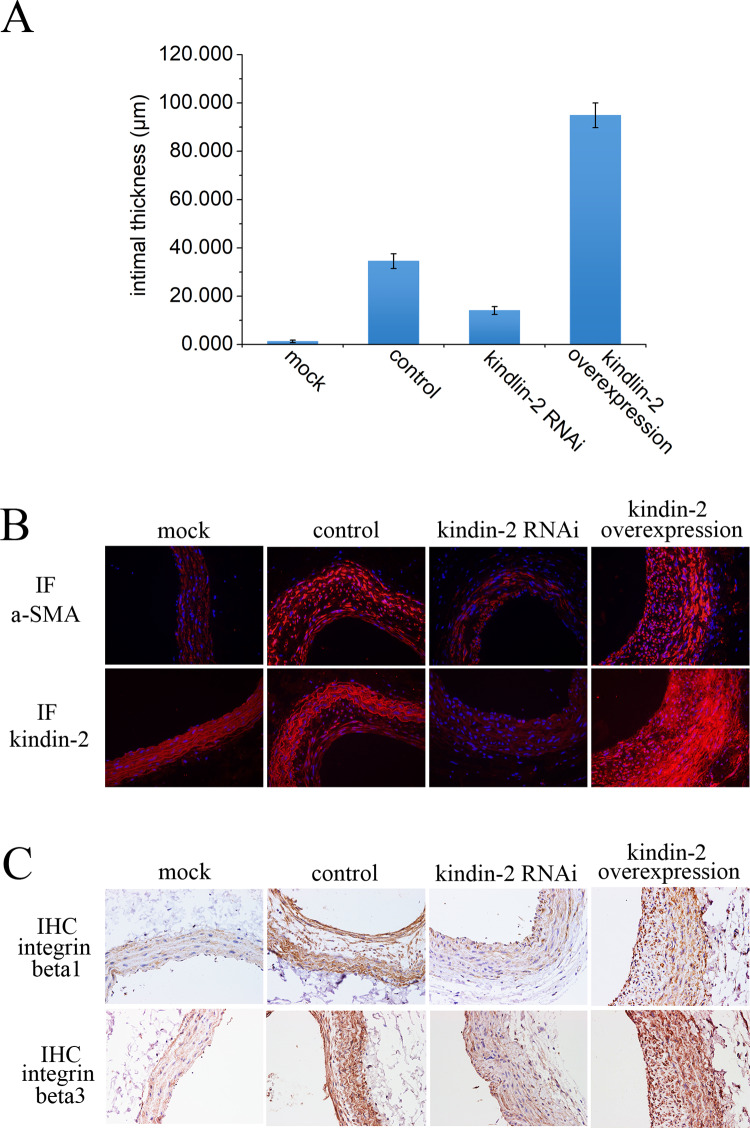
In rat model, Kindlin-2 improves vascular hyperplasia through integrinβ1 andβ3. (A) The effect of Kindlin-2 on the changes of intimal thickness in rats with vascular hyperplasia. Kindlin-2 RNAi or overexpressed adenovirus were injected into the injured vascular hyperplasia model of rats, and the changes of intimal thickness were identified by HE staining. Kindlin-2 RNAi or overexpression can inhibit or promote vascular proliferation in rat models. (B) Immunofluorescence was used to determine the effect of adenovirus injected on Kindlin-2 expression in vivo. Immunofluorescence staining showed that the injected adenovirus could inhibit or overexpress Kindlin-2 in vivo. (C) The expression of integrinβ1 andβ3 was detected by immunohistochemistry. The adenovirus injected can inhibit or promote the expression of integrinβ1 andβ3f through Kindlin-2.

In vivo, because cells are in different microenvironments, sometimes the results obtained from cell experiments do not represent the real situation in the vivo. Therefore, we decided to further validate the role and mechanism of Kindlin-2 in the rat model of vascular traumatic hyperplasia. We first constructed a rat model of vascular injury and hyperplasia as described previously. After that, HE staining was performed on rat vascular smooth muscle. After staining, we analyzed the ratio of intima to adventitia. The results showed that the ratio of intima to adventitia in the animal model group was significantly higher than that in the control group. Overexpression of Kindlin-2 could aggravate this process, and inhibition of Kindlin-2 could slow down this phenomenon (Fig 5A). Then we detected the expression of Kindlin-2, a-SMA in VSMCs by immunofluorescence. We found that the overexpression of Kindlin-2 promoted intimal hyperplasia, which was caused by the proliferation or migration of VSMCs, compared with the control group and the mock group (Fig 5B). At the same time, we also found that the expression of integrinβ1 andβ3 was upregulated by immunohistochemistry, which indicated that Kindlin-2 regulated the proliferation and migration of VSMC in vivo (Fig 5C). Therefore, we have demonstrated by immunofluorescence and immunohistochemistry that Kindlin-2 can also promote the proliferation and migration of VSMCs by regulating integrin expression in vivo.

## Discussion

Vascular hyperplasia after vascular injury is one of the typical symptoms of vascular injury [14]. In previous studies, researchers have identified many factors related to vascular hyperplasia [15–18]. In particular, factors related to proliferation and migration of vascular smooth muscle cells, such as integrinβ1 andβ3.

In recent years, with the further development of basic medical research, more and more gene functions have been explained. In cancer, Kindlin-2 has been shown to promote the proliferation and migration of cancer cells through AKT and FAK signaling pathways [9,11]. At the same time, some studies have shown that Kindlin-2 can promote the phosphorylation of integrinβ1 andβ3, thereby enhancing the activity of integrinβ1 andβ3 [12]. However, the role of Kindlin-2 in vascular hyperplasia has not been studied.

In this study, we first demonstrated that Kindlin-2 promotes VSMCs proliferation through FAK and AKT pathways at the cellular level by constructing knockdown and overexpression adenoviruses of Kindlin-2. Inhibition of Kindlin-2, or AKT and FAK, can promote the apoptosis of VSMCs by inhibiting integrinβ1 andβ3 activity. Then, to further confirm the role of Kindlin-2 in vivo, we constructed a rat model of vascular hyperplasia after vascular injury. In vivo, we also observed that Kindlin-2 could promote the expression and activity of integrinβ1 andβ3, and lead to an increase in the proportion of vascular intima, which greatly aggravated vascular hyperplasia in rats. This proves that Kindlin-2 also affects integrinβ1 andβ3 activity through FAK and AKT signaling pathways in rats to influence the process of vascular proliferation.

Although our study preliminarily confirmed the role and mechanism of Kindlin-2 in the process of angiogenesis, we need to further explore how Kindlin-2 affects the phosphorylation of FAK and AKT. In addition, the subjects of this experiment are all rats. Whether Kindlin-2 has the same effect in the process of human vascular proliferation also needs further clarification.

In conclusion, we have demonstrated ex vivo and in vivo that Kindlin-2 can affect integrinβ1 andβ3 activity through FAK and AKT signaling pathways, thereby affecting the process of vascular proliferation, suggesting that Kindlin-2 may be a potential target for the treatment of post-traumatic vascular hyperplasia.

## Supporting information

S1 File (ZIP)
