# Supplementary figures and images for: Effects of Kindlin-2 on proliferation and migration of VSMC and integrinβ1 andβ3 activity via FAK-PI3K signaling pathway
Source: PLoS One. 2020 Jun 30;15(6):e0225173. doi: 10.1371/journal.pone.0225173 (PMC7326154; doi:10.1371/journal.pone.0225173)

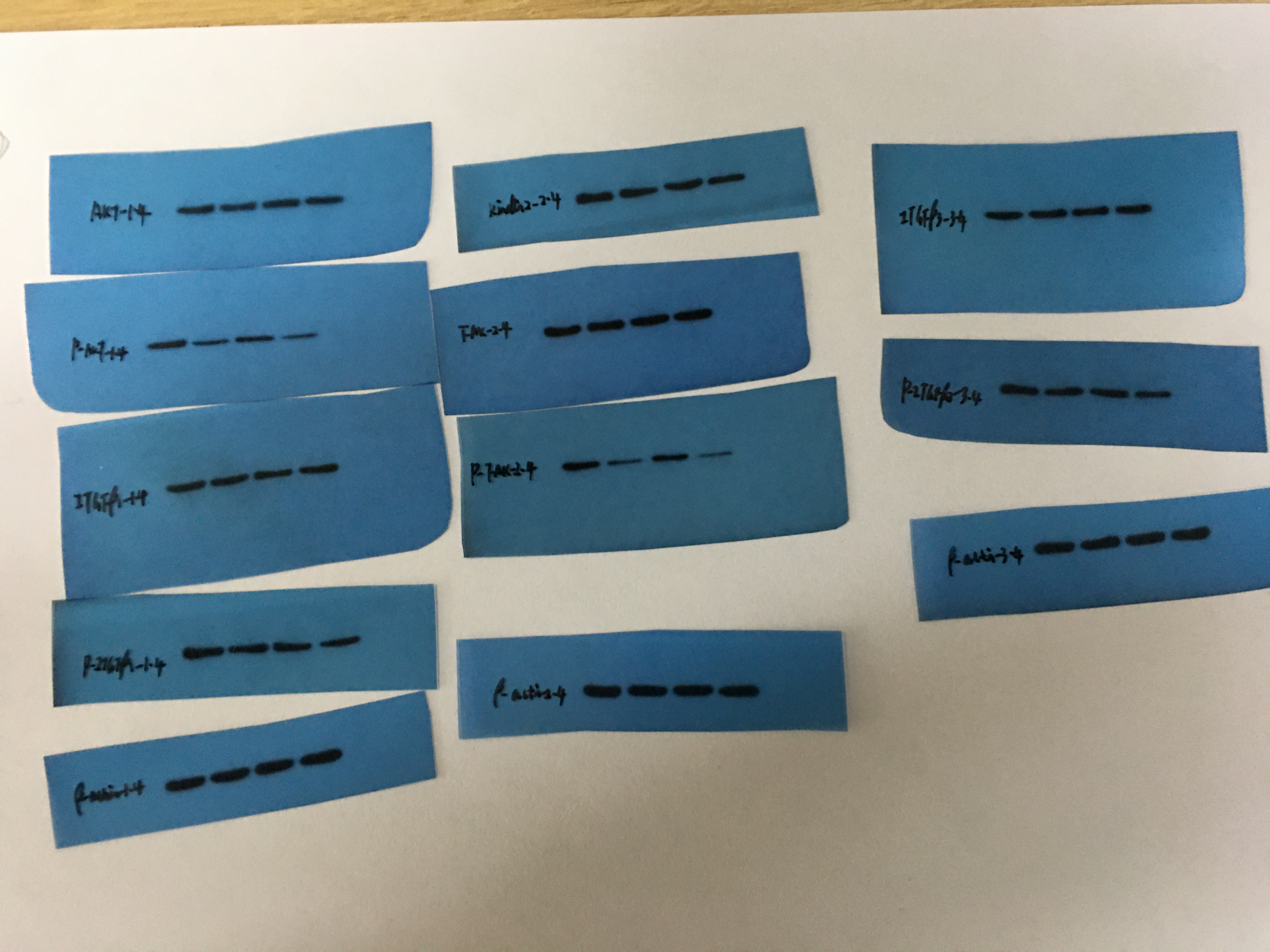

Supplement: S1 File — (ZIP) [file pone.0225173.s001.zip › Western blot∩╝êsource data∩╝ë/Figure 4D∩╝êsource data∩╝ë.JPG]

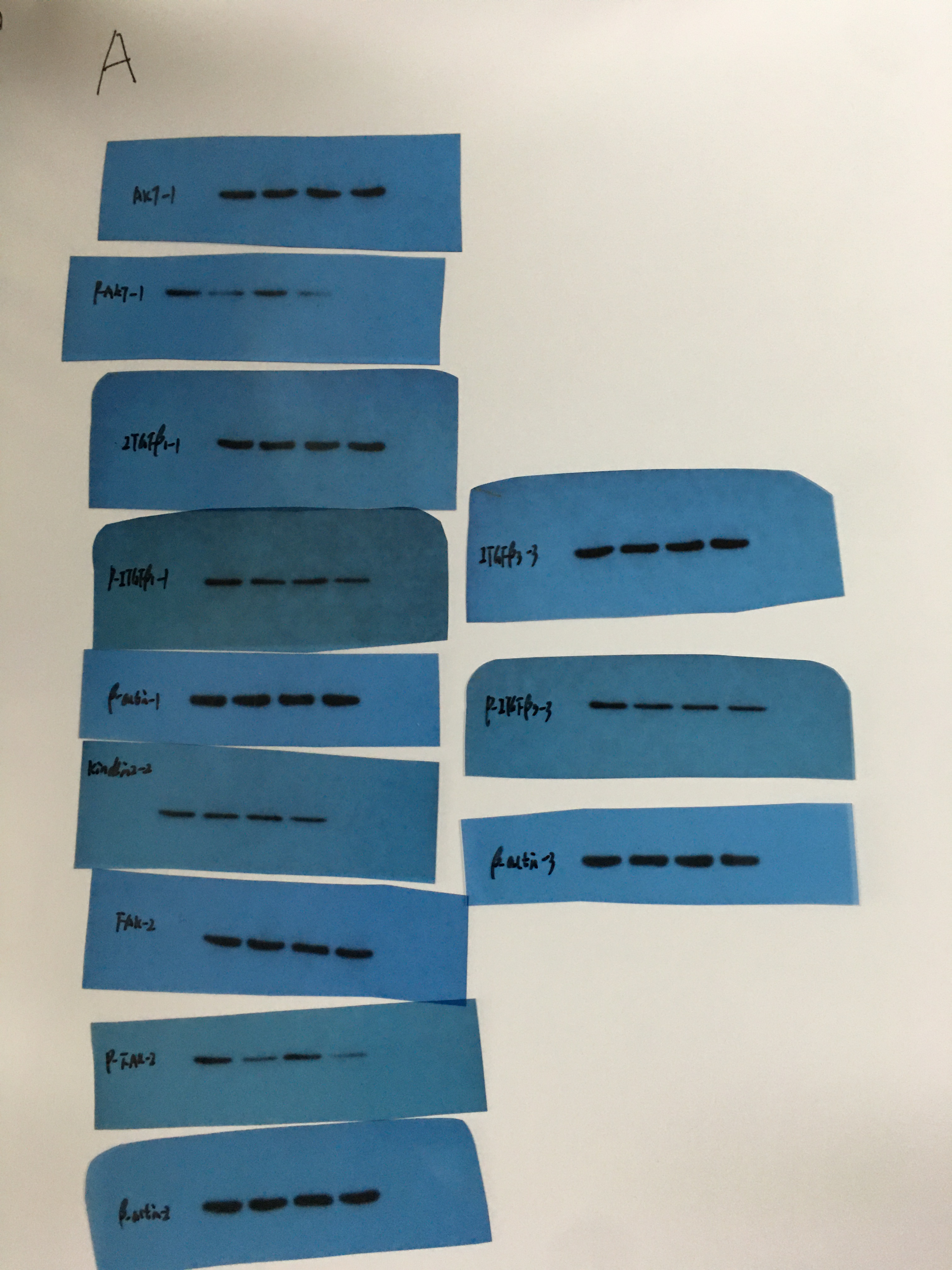

Supplement: S1 File — (ZIP) [file pone.0225173.s001.zip › Western blot∩╝êsource data∩╝ë/Figure 4A∩╝êsource data∩╝ë.JPG]

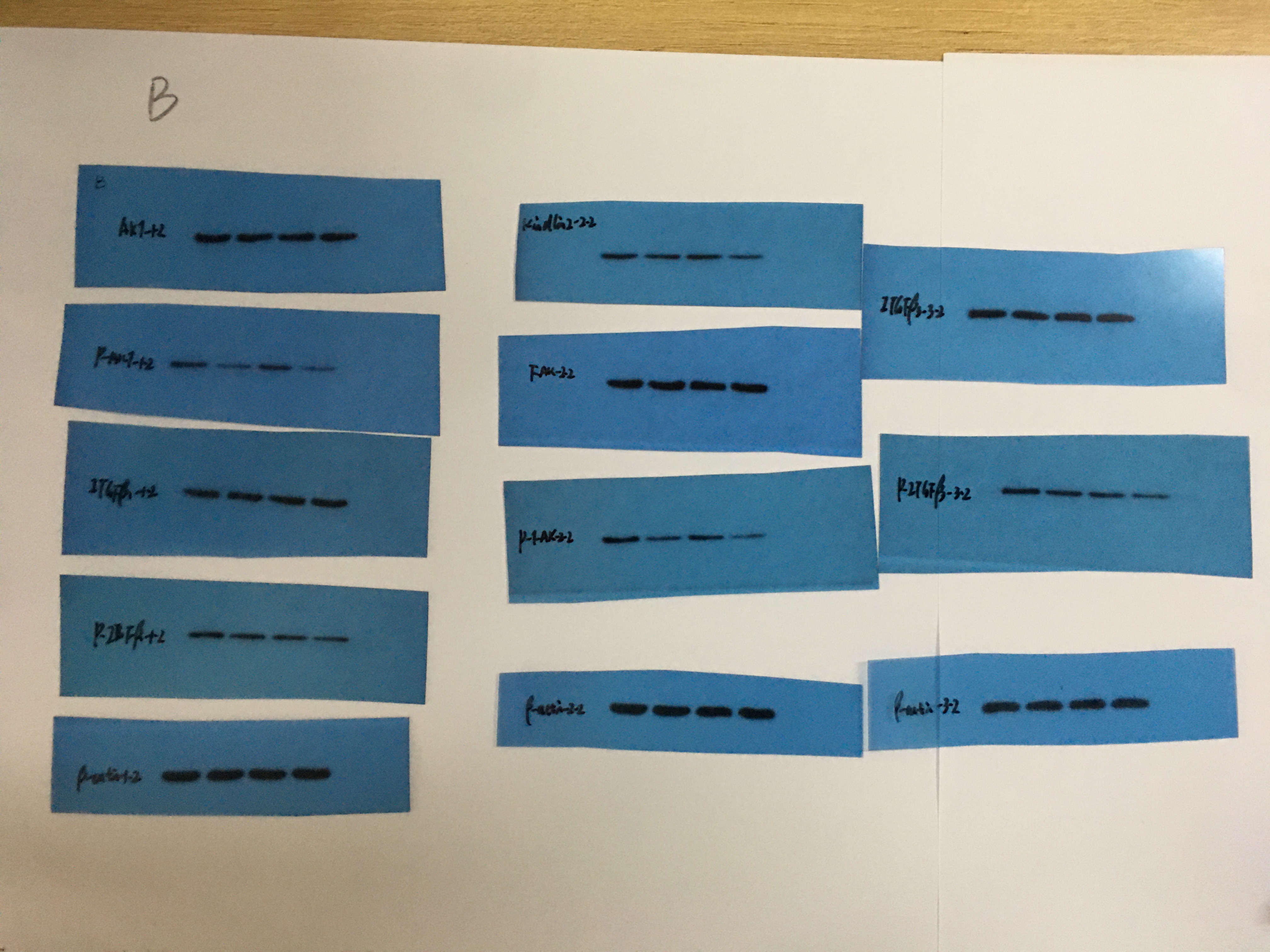

Supplement: S1 File — (ZIP) [file pone.0225173.s001.zip › Western blot∩╝êsource data∩╝ë/Figure 4B∩╝êsource data∩╝ë.JPG]

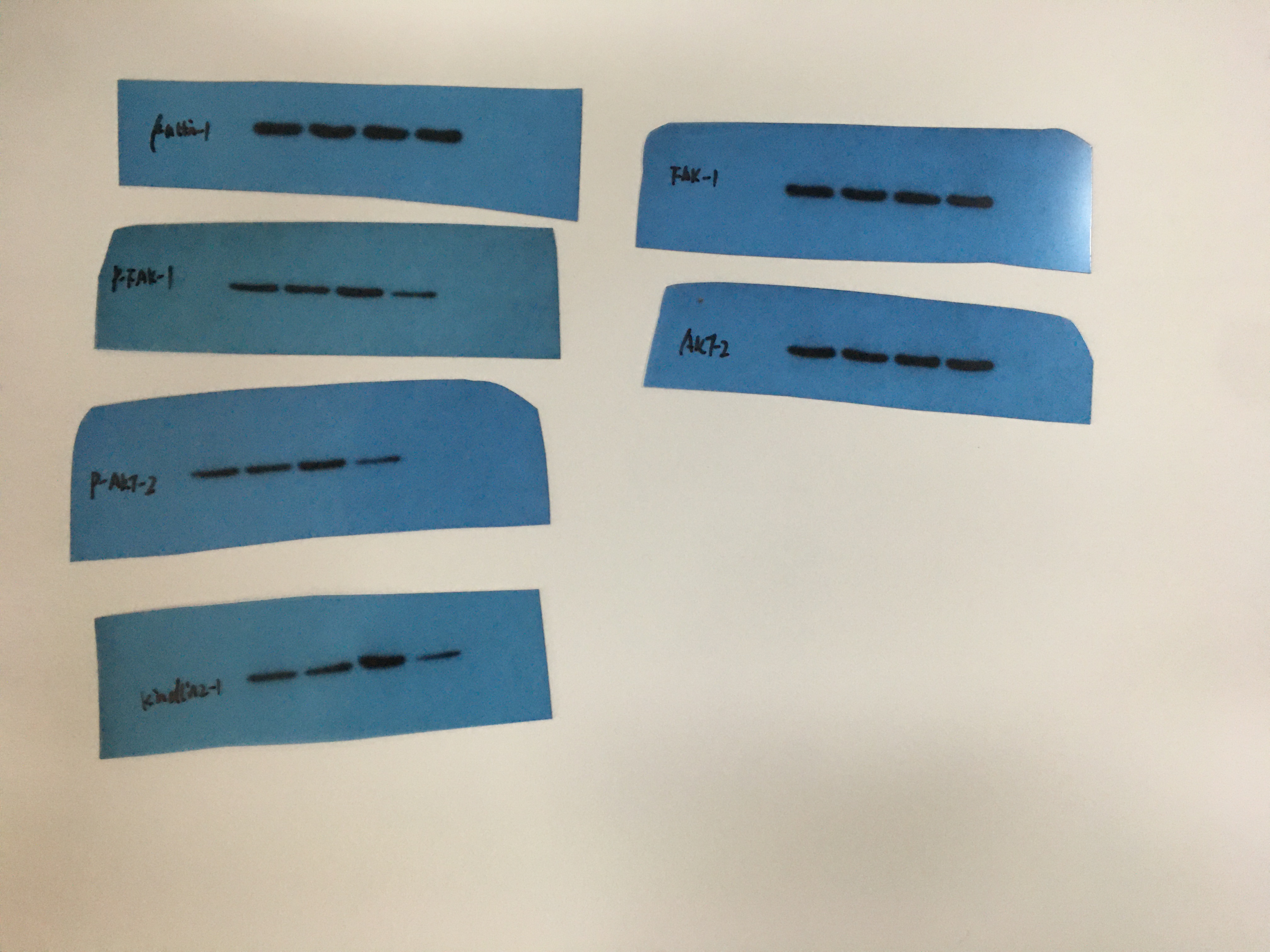

Supplement: S1 File — (ZIP) [file pone.0225173.s001.zip › Western blot∩╝êsource data∩╝ë/Figure 1B∩╝êsource data∩╝ë.JPG]

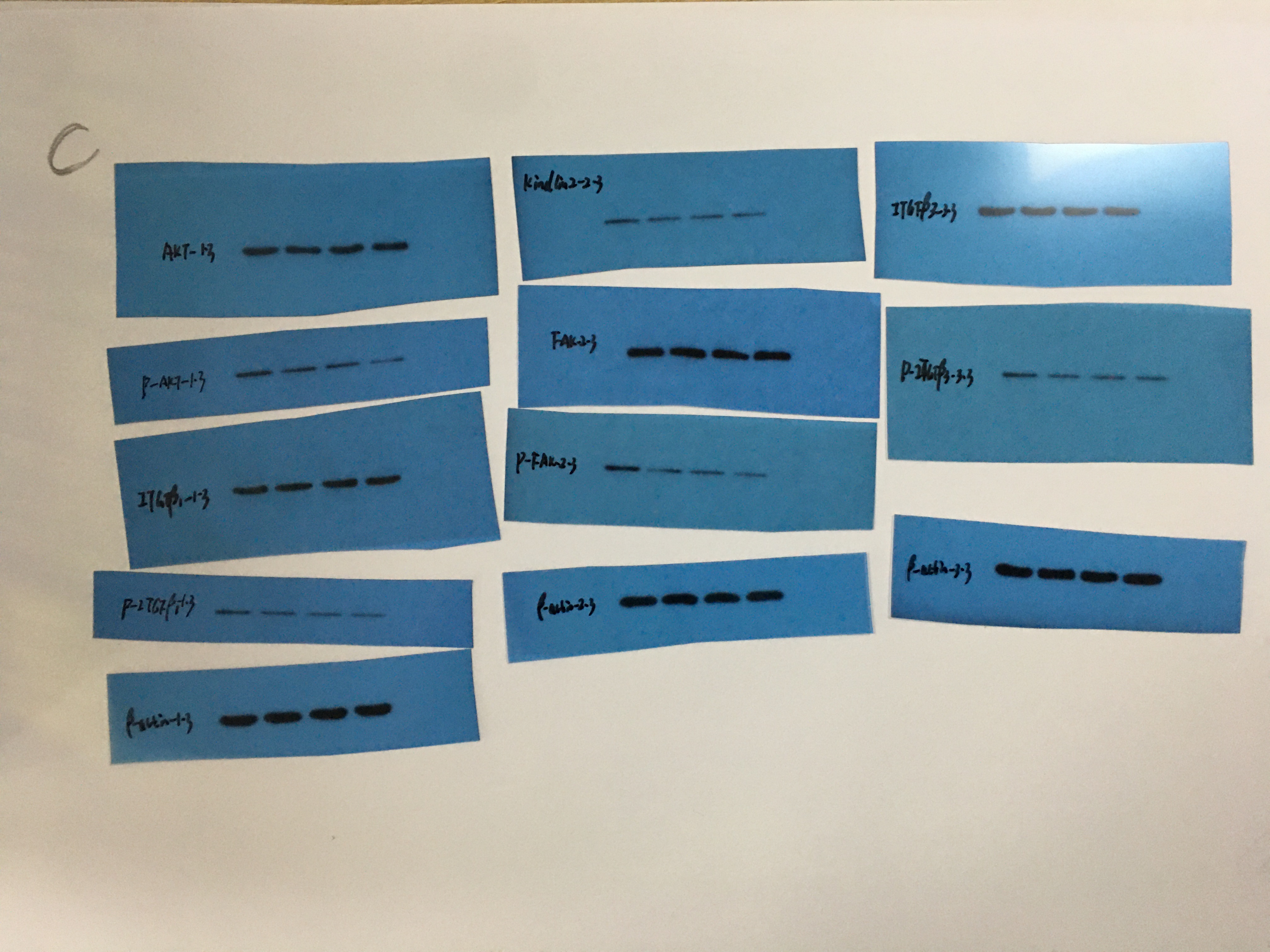

Supplement: S1 File — (ZIP) [file pone.0225173.s001.zip › Western blot∩╝êsource data∩╝ë/Figure 4C∩╝êsource data∩╝ë.JPG]
